# Preoperative Antihypertensive Medication in Relation to Postoperative Atrial Fibrillation in Patients Undergoing Cardiac Surgery: A Meta-Analysis

**DOI:** 10.1155/2017/1203538

**Published:** 2017-02-13

**Authors:** Ai-Guo Zhou, Xian-Xue Wang, Dao-Bo Pan, An-ji Chen, Xiong-fei Zhang, Hui-wei Deng

**Affiliations:** Department of Anesthesiology of the First People's Hospital of Changde City, Changde, Hunan, China

## Abstract

*Background*. We undertake a systematic review and meta-analysis to evaluate the effect of preoperative hypertension and preoperative antihypertensive medication to postoperative atrial fibrillation (POAF) in patients undergoing cardiac surgery.* Methods*. We searched PubMed, Embase, and Cochrane Library (from inception to March 2016) for eligible studies. The outcomes were the effects of preoperative hypertension, preoperative calcium antagonists regimen, preoperative ACE inhibitors regimen, and preoperative beta blocking agents regimen with POAF. We calculated pooled risk ratios (OR) and 95% CIs using random- or fixed-effects models.* Results*. Twenty-five trials involving 130087 patients were listed. Meta-analysis showed that the number of preoperative hypertension patients in POAF group was significantly higher (*P* < 0.05), while we found that there are no significant differences between two groups in Asia patients by subgroup analysis, which is in contrast to other outcomes. Compared with the Non-POAF group, the number of patients who used calcium antagonists and ACE inhibitors preoperatively in POAF group was significantly higher (*P* < 0.05). And we found that there were no significant differences between two groups of preoperative beta blocking agents used (*P* = 0.08).* Conclusions*. Preoperative hypertension and preoperative antihypertensive medication in patients undergoing cardiac operations seem to be associated with higher risk of POAF.

## 1. Introduction

Atrial fibrillation (AF) is a supraventricular tachyarrhythmia characterized by uncoordinated atrial activation with deterioration of mechanical function [1]. Postoperative atrial fibrillation (POAF) is the most common perioperative cardiac arrhythmia [2]. Although many studies have tried to assess risk factors for POAF, it remains incomplete and unclear. Moreover, patients with POAF have increased risk of stroke, other arrhythmias, cardiovascular mortality, and higher health-care costs compared with patients without POAF [3–5]. Constantly ascertaining the risk factors and prevention of POAF is of great importance for the physician.

Patient characteristics resulting in POAF are many. Some studies have confirmed that preoperative hypertension is a danger factor to POAF in patients with cardiac surgery [6–8], while a lot of studies suggestion that the number of preoperative hypertension patients has not significantly statistical differences between POAF group and Non-POAF group [9, 10]. So we undertook a systematic review and meta-analysis to evaluate the effect of preoperative hypertension to POAF in patients with cardiac surgery.

## 2. Methods

This systematic review was performed according to the guidelines of the preferred reporting items for systematic reviews and meta-analyses (PRISMA) [6]. We prospectively registered our system review at PROSPERO (Registration number: CRD42016038101). The proposed study will utilize published data; as such there is no need for ethical approval.

### 2.1. Data Sources and Search Strategy

The PubMed, Embase, and Cochrane Library databases were searched from inception to February 2016 for relevant studies investigating the association between preoperation hypertension and POAF in cardiac surgical patients. The following search terms were used: Atrial fibrillation, “Fibrillation, atrial”, Familial atrial fibrillation, Auricular fibrillation, “Fibrillation, auricular”, Postoperative period, “Period, postoperative”, Cardiac surgical procedures, “Procedure, cardiac surgical”, “Surgical procedure, cardiac”, “Surgical procedures, heart”, Cardiac surgical procedure, Heart surgical procedures, “Procedure, heart surgical”, “Surgical procedure, heart”. A manual search of the reference sections of included trials, published meta-analyses, and relevant review articles was conducted to identify additional articles. If duplicated data were shown in several studies, only the most recent, largest, or most complete study was included.

Original studies included in our meta-analysis had to meet the following criteria: (1), an observation human study; (2) investigating the association between preoperative hypertension and POAF in cardiac surgical patients; (3) providing sufficient data to calculate them. Only English language studies were chosen.

### 2.2. Data Extraction and Assessment of Study Quality

Patient characteristics (authors, number of patients, year of publication, ASA rating, age, gender, type of surgery and anaesthesia, and endpoint) were recorded. If the data mentioned above were unavailable in the article, the corresponding authors were called upon for missing information. All of the data were independently extracted using a standard data collection form by both authors, and then the collected data were checked and entered into Review Manager analysis software (RevMan) Version 5.3. All discrepancies were checked, and a consensus was achieved by discussion. A record of reasons for excluding studies was kept.

The order from higher to lower in the quality of studies was the following: (1) prospective cohort study, (2) retrospective cohort study, and (3) case-control study. We evaluate the quality of the studies by Newcastle-Ottawa Scale (NOS) [11]: a maximum of nine points to each cohort study (four for quality of selection, two for comparability, and three for quality of outcome and adequacy of follow-up) and a score of nine points to every case-control study (four for quality of selection, two for comparability, and three for quality of exposure). The score of each study less than 6 was regarded as a low-quality study; otherwise, it was a high-quality study.

### 2.3. Statistical Analysis

The risk ratio (RR) with 95% CI was used as a common measure of the effect between the two groups. The meta-analysis was carried out using Review Manager, version 5.3 (The Cochrane Collaboration, Software Update, Oxford, UK). Statistical heterogeneity across studies was usually investigated using the *I*^2^ statistic. When *I*^2^ values of less than 50% were determined, heterogeneity could be accepted, and the fixed-effects model was expected to be adopted. Otherwise, the randomized-effects model was adopted, and we investigated the influence of a single study on the overall pooled estimate by omitting one study in each turn. A *P* value of <0.05 was considered statistically significant.

To explore potential sources of heterogeneity among studies, we performed four sets of subgroup analysis: by study design (cohort versus case-control studies), by type of cardiac operation performed (coronary artery bypass grafting (CABG) only versus aortic valve replacement (AVR) only), by NOS scores (less than 6 versus with or higher 6), and by different region (Asia versus Europe versus America versus Oceania). We also performed a sensitivity analysis by excluding studies where the association between hypertension and POAF was opposite to the one from others.

## 3. Results

### 3.1. Identification of Eligible Studies

**Figure 1:**
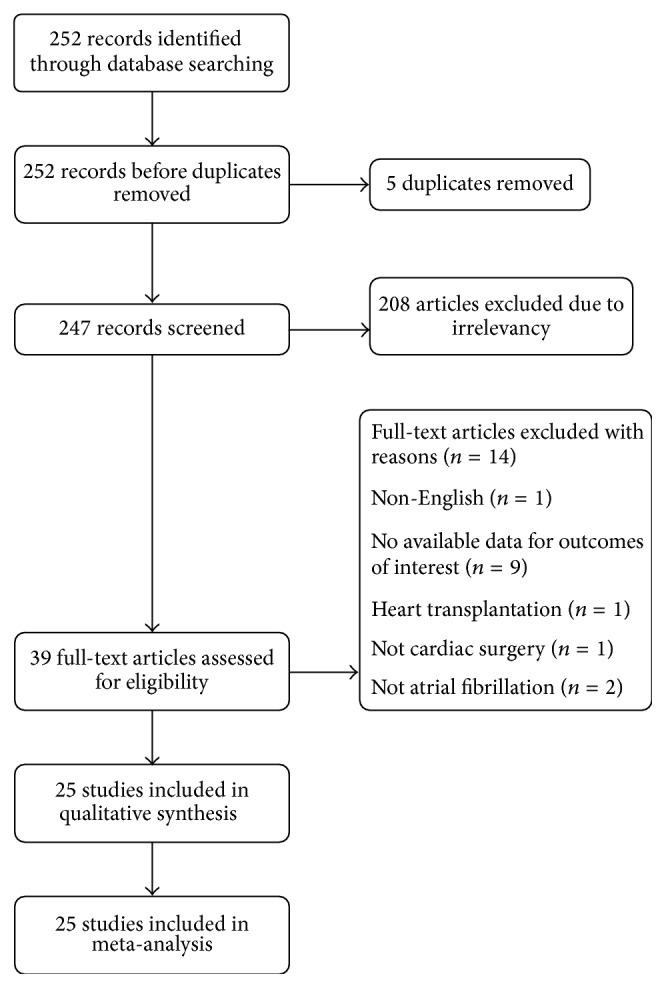
Flow diagram of search strategy and study selection.

In total, 252 potentially relevant abstracts were identified. After duplicates were removed, 247 unique abstracts remained. After examining the abstracts, 39 publications seemed to meet the inclusion criteria. Of these, 14 were excluded for the following reasons: Non-English language [32], no available data on the outcome of interest in [33–41], heart transplantation [42], no cardiac surgery [43], and no atrial fibrillation [44, 45]. Finally, the remaining 25 studies [6–10, 12–31] to existing data met our selection criteria and were included in the systematic review. A flow diagram of the search strategy and study selection is illustrated in Figure 1.

### 3.2. Study Characteristics

The characteristics of all included studies were presented in Table 1. Most patients underwent CABG only; three studies reported Aortic valve replacements alone [14, 24, 30]. The type of cardiac operation in five studies contained CABG and valve operation [6, 13, 15, 22, 23]. These studies were published between 1997 and 2014. All studies clearly indicated the study population and defined the outcome. Sample size of included studies varied from 53 to 49264.

Eleven studies were cohorts [8, 10, 13, 23, 24, 26–31] and the rest were case-control studies [6, 7, 9, 12, 14–22, 25]. The quality of the included studies was assessed by NOS score. High NOS score of the studies included was 20 and the mean score was 6.12 (range from 2 to 8). Quality assessment of the 25 studies was shown in Table 1. Three studies performed logistic regression analysis of preoperative drug administration for POAF in Table 2.

### 3.3. Meta-Analysis of Primary Outcomes

#### 3.3.1. The Effect of Preoperative Hypertension to POAF

**Figure 2:**
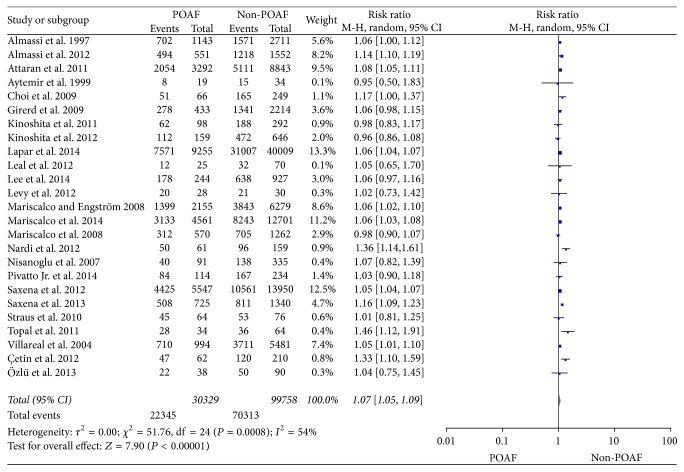
The effect of preoperative hypertension to POAF.

**Figure 3:**
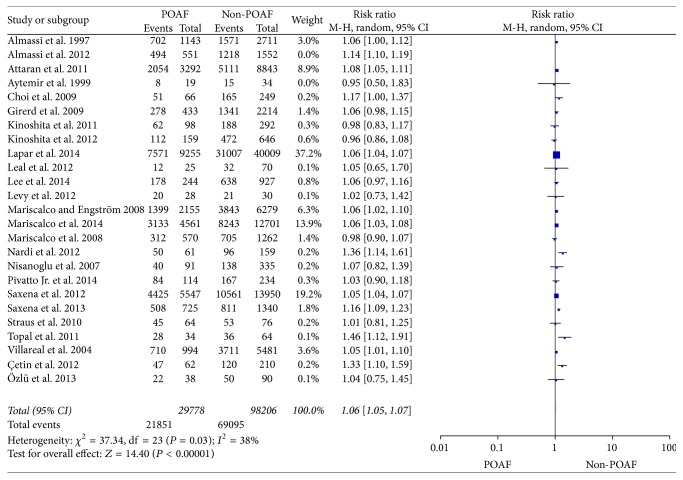
The effect of preoperative hypertension to POAF by sensitivity analysis.

The aggregated results were studied in 25 trials [6–10, 12–31] and illustrated in Figure 2. Heterogeneity was noted among the studies (*I*^2^ = 54%; *P* = 0.0008), and a randomized-effects model was chosen. The results indicate that the number of preoperative hypertension patients in POAF group was significantly higher than Non-POAF group (RR = 1.07, 95% CI: 1.05–1.09, *P* < 0.00001). After investigating the influence of a single study on the overall pooled estimate by omitting one study in each turn, we found that the *I*^2^ is still higher than 50 except for one study [8]. When we omit the study of Almassi's [8], the *I*^2^ drop to 38% and a fixed-effects model was selected, and the outcome between two groups has a significant difference (RR = 1.06, 95% CI: 1.05–1.07, *P* < 0.00001) (Figure 3).

#### 3.3.2. Subgroup Analysis between Preoperative Hypertension and POAF

We performed subgroup analysis among studies to further demonstrate the relations of preoperative hypertension and POAF and explore potential sources of heterogeneity, while heterogeneity still existed (Table 3). Dividing the different regions that studies come from, statistically significant relations were observed for Europe (RR = 1.08; 95% CI: 1.04–1.12; *P* < 0.0001) and America (RR = 1.07; 95% CI: 1.04–1.11; *P* < 0.00001), while there were no significant difference in Asia (RR = 1.03; 95% CI: 0.97–1.09; *P* = 0.32) and Oceania regions (RR = 1.10; 95% CI: 1.00–1.21; *P* = 0.05). By observing the different study design, we found that the preoperative hypertension was significantly associated with POAF in cohort studies (RR = 1.11; 95% CI: 1.05–1.17; *P* = 0.0002) and case-control studies (RR = 1.06; 95% CI: 1.05–1.07; *P* < 0.00001). Dividing the studies into the high-quality and low-quality, statistically significant relations were observed for less than 6 (RR = 1.13; 95% CI: 1.01–1.26; *P* = 0.03) and higher or with 6 (RR = 1.07; 95% CI: 1.05–1.09; *P* < 0.00001). The preoperative hypertension was also significantly associated with POAF in different type of cardiac operation performed, CABG alone (RR = 1.07; 95% CI: 1.05–1.09; *P* < 0.00001), and AVR only (RR = 1.13; 95% CI: 1.07–1.20; *P* < 0.0001) (Table 3).

#### 3.3.3. Preoperative Calcium Antagonists Regimen with POAF

**Figure 4:**
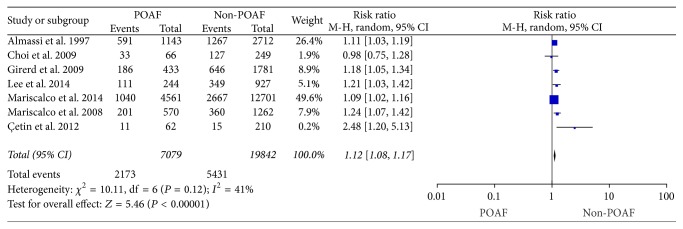
The effect of preoperative calcium antagonists regimen to POAF.

Seven studies [6, 12, 13, 16, 23, 26, 29] with a total of 26921 patients reported preoperative calcium antagonists applied association with POAF. Heterogeneity among studies could be accepted (*I*^2^ = 41%; *P* = 0.12), and a fixed-effects model was selected. Compared with the Non-POAF group, the number of patients who used calcium antagonists in POAF group was significantly greater (RR: 1.12, 95% CI: 1.08–1.17, *P* < 0.00001) (Figure 4).

#### 3.3.4. Preoperative ACE Inhibitors Regimen with POAF

**Figure 5:**
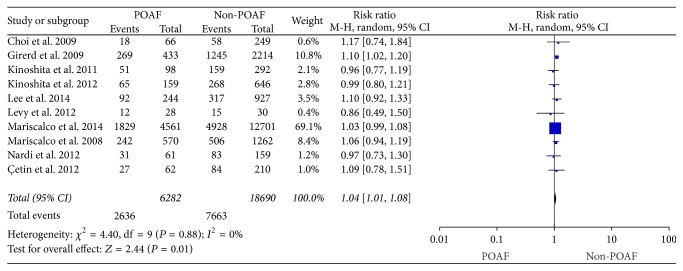
The effect of preoperative ACE inhibitors regimen to POAF.

Ten studies [6, 12, 13, 16, 17, 20, 26, 27, 29, 30] compared the preoperative ACE inhibitors used between two groups. There were no heterogeneity among the studies (*I*^2^ = 0%; *P* = 0.88), and a fixed-effects model was chosen. After integrating the data, people who applied ACE inhibitors before operation were significantly greater in POAF group when compared with Non-POAF group (RR: 1.04, 95% CI: 1.01–1.08, *P* = 0.01) (Figure 5).

#### 3.3.5. Preoperative Beta Blocking Agents Regimen with POAF

**Figure 6:**
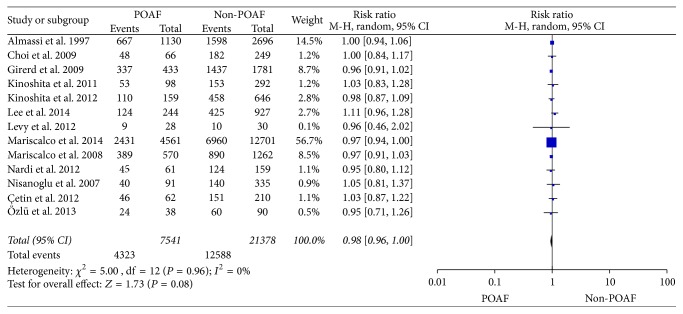
The effect of preoperative beta blocking agents regimen to POAF.

Thirteen studies [6, 9, 12, 13, 16, 17, 20, 23, 26–30] compared the preoperative beta blocking agents used in the POAF group and Non-POAF group. There was no heterogeneity among the studies (*I*^2^ = 0%; *P* = 0.96), and a fixed-effects model was chosen. After examining the studies by meta-analysis, we found that there were not significant difference between two groups of preoperative beta blocking agents used (RR: 0.98, 95% CI: 0.96–1.00, *P* = 0.08) (Figure 6).

## 4. Discussion

This is the first time meta-analysis to discuss the effect of preoperative hypertension to POAF. The pooled meta-analysis of 25 studies suggested that patients who have hypertension before operation were easier to develop AF postoperative. Preoperative ACE inhibitors and calcium antagonists regimen may be risk factors for POAF in patients undergoing cardiac surgery. In addition, patients with preoperative beta blocking agents were not linked with POAF.

People with hypertension are liable to suffer left atrial enlargement by the increased cardiac afterload, which was leading to atrial remodeling following the progression of disease [46]. Hypertension caused left ventricular hypertrophy and increases left ventricular stiffness, decreases coronary flow reserve, wall stress, and filling pressure and increases the activation of the sympathetic nervous system, which are associated with AF occurrence. At the same time, the proliferation and differentiation of fibroblasts into myofibroblasts cause disturbances in extracellular matrix. Studies suggested that the cardiac extracellular matrix remodeling was significantly changed in the hypertensive patients with AF [47, 48]. This may help explain why we found the numbers of preoperative hypertension patients in POAF group to be larger than Non-POAF group (*P* < 0.00001). Following consideration of the heterogeneity, we performed sensitivity analysis and subgroup analysis to talk about the potential reasons. We performed four sets of subgroup analysis and found the heterogeneity still exists in some groups (Table 2). We found that there is no significant difference between two groups in Asia patients with subgroup analysis, which is in contrast to other outcomes. Race factor may contribute to this result, while only four studies come from the Asia region and more studies are needed to confirm it. Then we omit the study of Almassi et al.'s [8] from 25 literatures; the *I*^2^ drop to 38% and is accepted. Almassi compared the rate of POAF between on- and off-pump coronary artery bypass and found preoperative hypertension is a significant factor to POAF in on-pump coronary artery bypass group. Studies have confirmed a lower incidence of POAF in off-pump coronary artery bypass patients [49, 50]. Other studies in our meta-analysis did not state the type of CABG similar to Almassi's study, which may be the cause of heterogeneity.

Antihypertensive drugs are prescribed mainly to reduce the morbidity and mortality caused by hypertension and its complications, while we observed that preoperative application of calcium antagonists and ACE inhibitors regimens is meaningful risk to POAF (*P* < 0.05). Read through those articles and attempt to find the confounding factors that influence the outcomes. Most of the studies did not perform logistic regression analysis except three studies [16, 27, 29]. Although *P* values in three studies are all greater than 0.05, the OR values in two studies [16, 29] are greater than 1 (Table 2). It may be telling us that calcium antagonists and ACE inhibitors regimens have a positive effect on POAF, in spite of no significant difference existing. Several reasons contribute to the outcome: (1) preoperative application of calcium antagonists and ACE inhibitors regimens is significant risk factors to POAF and needs more studies to confirm it; (2) some confounding factors affect the results and need further analysis; (3) the myocardial excitability is higher in patients who apply calcium antagonists and ACE inhibitors preoperatively, for they usually stop these drugs postoperatively.

We recognize several limitations in our analysis. First, we performed an unavailable meta-analysis, and no access to individual patient data from individual studies was available. Second, this was a meta-analysis of observational studies. Subgroup analyses for some potential confounding of the association between preoperative hypertension and POAF were performed, and we did not find any strong subgroup effects. Third, none of the studies reported the association of different level hypertension in POAF in cardiac surgery patients, and therefore, we could not analyze this. Finally, this meta-analysis was based on studies published in the English language and unpublished literature could be missing, which may have generated bias.

## 5. Conclusion

Preoperative hypertension in patients undergoing cardiac operations seems to be associated with higher risk of POAF. Considering the limitations of this study, our finding should be reviewed with caution, and large-scale studies are needed to confirm our findings.

## Figures and Tables

**Table 1:** Characteristics of the 25 studies included in the meta-analysis.

Study	Number of patients (AF/non-AF)	Country	Study design	Style of operation	OR (95% CI)	Definition of AF	NOS points
Lee et al. 2014	244/927	Korea	Case-control	CABG	NA	Postoperative atrial fibrillation was defined as newly developed AF documented by electrocardiography (ECG) or continuous monitoring during the first 10 days after surgery	8

Mariscalco and Engström 2008	2155/6279	Sweden	Case-control	Cardiac surgery	NA	The AF definition included arrhythmia successfully treated as well as those persistent at discharge. The arrhythmia, as defined by physician assessment, was on the basis of a telemetry strip or from a 12-lead electrocardiogram recording	7

Pivatto Jr. et al. 2014	114/234	Brazil	Case-control	AVR	NA	AF consisted of any episode of supraventricular arrhythmia whose electrocardiography tracing showed “f” waves with varying morphology and amplitude as well as irregular ventricular rhythm	4

Attaran et al. 2011	3292/8843	United Kingdom	Case-control	Cardiac surgery	NA	AF, confirmed on electrocardiogram (ECG) for any length of time	8

Girerd et al. 2009	433/2214	Canada	Case-control	CABG	Hypertension: OR = 0.89 (0.68 to 1.16) Preoperative drugs: beta blockers: OR = 1.03 (0.77 to 1.39) ACEI and/or ARBs: OR = 1.26 (0.98 to 1.61) Calcium channel-blockers: OR = 1.18 (0.92 to 1.52)	AF was defined as any sustained episode recorded during the postoperative hospital stay and requiring medical and/or electrical cardioversion	6

Kinoshita et al. 2012	159/646	Japan	Case-control	CABG	Hypertension: OR = 0.85 (0.58 to 1.18) Preoperative drugs: beta blockers: OR = 0.91 (0.61 to 1.41) ACEI and/or ARBs: OR = 0.93 (0.60 to 1.44)	The endpoint was new-onset AF after surgery, which was diagnosed when there was an irregular cardiac rhythm without p waves lasting more than 60 min that required further administration of antiarrhythmics, cardioversion, or anticoagulation 8 therapy	6

Villareal et al. 2004	994/5481	United States	Case-control	CABG	NA	Postoperative AF was defined by the documentation of AF of any duration at any time in the postoperative period on a physician assessment, on the basis of a rhythm strip or 12-lead electrocardiogram recording	8

Topal and Eren 2011	34/64	Turkey	Case-control	CABG	NA	NA	4

Kinoshita et al. 2011	98/292	Japan	Case-control	CABG	Hypertension: OR = 0.92 (0.59 to 1.43) Preoperative drugs: beta blockers: OR = 0.79 (0.44 to 1.28) ACEI and/or ARBs: OR = 0.90 (0.59 to 1.38)	The endpoint was new-onset AF after operation, which was diagnosed when there was an irregular cardiac rhythm without p waves lasting more than 60 minutes	6

Saxena et al. 2012	5547/13950	Australia	Case-control	CABG	NA	POAF was defined as evidence of new AF that required treatment by electrocardiography or continuous monitoring during the postoperative period	7

Lapar et al. 2014	9255/40009	United States	Case-control	Cardiac surgery	NA	NA	7

Almassi et al. 2012	551/1552	United States	Cohort	CABG	Hypertension: OR = 1.76 (1.23 to 2.50)	AF was defined as any abnormal atrially originated irregular rhythm lasting more than 30 minutes	7

Almassi et al. 1997	1143/2712	United States	Cohort	Cardiac Surgery	NA	NA	6

Mariscalco et al. 2008	570/1262	Italy	Cohort	CABG	NA	NA	7

Saxena et al. 2013	725/1340	Australia	Cohort	AVR	NA	POAF was defined as evidence of new AF that required treatment and was discovered by electrocardiography or continuous monitoring during the postoperative period.	8

Leal et al. 2012	25/70	Brazil	Case-control	CABG	NA	We defined AF occurrence as any AF episode requiring any type of medical treatment and/or lasting for more than 20 min within the hospital stay period	6

Choi et al. 2009	66/249	Korea	Cohort	CABG	NA	NA	4

Nardi et al. 2012	61/159	Italy	Cohort	CABG	Hypertension: OR = 1.71 (0.89 to 2.26) Preoperative drugs: beta blockers: OR = 0.91 (0.33 to 20.46) ACEI and/or ARBs: OR = 0.85 (0.33 to 20.17)	POAF, defined as any evidence of new AF by electrocardiography or continuous ECG monitoring, lasting at least 30 seconds during the postoperative period in our hospital	6

Özlü et al. 2013	38/90	Turkey	Cohort	CABG	NA	Presence of POAF lasting more than 5 min during hospitalization was detected by using continuous telemetry or 12-lead electrocardiography	4

Çetin et al. 2012	62/210	Turkey	Cohort	CABG	Hypertension: OR = 1.638 (0.728 to 3.687) Preoperative drugs: calcium channel-blockers: OR = 1.929 (0.627 to 5.935)	POAF was defined as any episode of atrial fibrillation within the hospital stay after CABG surgery	6

Levy et al. 2012	28/30	France	Cohort	AVR	NA	POAF combined paroxysmal and persistent AF. Paroxysmal AF was defined as self-terminating AF, usually within 48 hours. Persistent AF was defined as an AF episode that lasted longer than 7 days or required termination by cardioversion	7

Mariscalco et al. 2014	4561/12701	United Kingdom	Case-control	Cardiac surgery	NA	POAF was documented on the basis of a rhythm strip or 12-lead ECG as previously described	7

Aytemir et al. 1999	19/34	Turkey	Cohort	CABG	NA	NA	5

Nisanoglu et al. 2007	91/335	Turkey	Case-control	CABG	Hypertension: OR = 1.12 (0.70 to 1.79)	AF was diagnosed if 12-lead ECG showed rapid oscillations or fibrillatory p waves that varied in size, shape, and timing, associated with irregular QRS complexes. For this study, postoperative	7

Straus et al. 2010	64/76	Yugoslavia	Cohort	CABG	NA	NA	2

AF = atrial fibrillation; CI = confidence interval; NOS = Newcastle-Ottawa Scale; OR = odds ratio; NA = not available.

**Table 2:** Logistic regression analysis of preoperative medication for POAF.

Study	OR	95% CI	*P* level	Model of logistic regression analysis
Girerd et al 2009				Multivariable logistic regression analysis
Calcium channel-blockers	1.18	0.92–1.52	0.18	
ACE-inhibitors	1.26	0.98–1.61	0.07	
Nardi et al. 2012				Multivariable logistic regression analysis
ACE-inhibitors	0.85	0.33–20.17	0.74	
Çetin et al. 2012				Binary logistic regression analysis
Calcium channel-blockers	1.929	0.627–5.935	0.252	

**Table 3:** Subgroup analysis between preoperative hypertension and POAF.

Variable	Number of studies	RR (95% CI)	*I* ^2^	Effects models	*P* value
Different region					
Asia	4	1.03 (0.97–1.09)	32	Fixed effects models	0.32
Europe	12	1.08 (1.04–1.12)	53	Random effects models	<0.0001
America	7	1.07 (1.04–1.11)	61	Random effects models	<0.00001
Oceania	2	1.10 (1.00–1.21)	87	Random effects models	0.05
Study design					
Case-control	14	1.06 (1.05–1.07)	0	Fixed effects models	<0.00001
Cohort	11	1.11 (1.05–1.17)	61	Random effects models	0.0002
Study quality score					
NOS ≥ 6	20	1.07 (1.05–1.09)	57	Random effects models	<0.00001
NOS < 6	5	1.13 (1.01–1.26)	25	Fixed effects models	0.03
Style of operation					
CABG	17	1.07 (1.05–1.09)	53	Random effects models	<0.00001
AVR	3	1.13 (1.07–1.20)	23	Fixed effects models	<0.0001

## Abbreviations

RR: Risk ratios
CABG: Coronary artery bypass grafting
AVR: Aortic valve replacement
NOS: Newcastle-Ottawa Scale
POAF: Postoperative atrial fibrillation
AF: Atrial fibrillation.
